# Serum AMH in Physiology and Pathology of Male Gonads

**DOI:** 10.1155/2013/128907

**Published:** 2013-10-24

**Authors:** Ewa Matuszczak, Adam Hermanowicz, Marta Komarowska, Wojciech Debek

**Affiliations:** Pediatric Surgery Department, Medical University of Bialystok, Waszyngtona 17, 15-274 Bialystok, Poland

## Abstract

AMH is secreted by immature Sertoli cells (SC) and is responsible for the regression of Müllerian ducts in the male fetus as part of the sexual differentiation process. AMH is also involved in testicular development and function. AMHs are at their lowest levels in the first days after birth but increase after the first week, likely reflecting active SC proliferation. AMH rises rapidly in concentration in boys during the first month, reaching a peak level at about 6 months of age, and then slowly declines during childhood, falling to low levels in puberty. Basal and FSH-stimulated levels of AMH, might become a useful predictive marker of the spermatogenic response to gonadotropic treatment in young patients with hypogonadotropic hypogonadism. After puberty, AMH is released preferentially by the apical pole of the SC towards the lumen of the seminiferous tubules, resulting in higher concentrations in the seminal plasma than in the serum. Defects in AMH production and insensitivity to AMH due to receptor defects result in the persistent Müllerian duct syndrome. A measurable value of AMH in a boy with bilateral cryptorchidism is predictive of undescended testes, while an undetectable value is highly suggestive of anorchia or ovaries, as would be the case in girls with female pseudohermaphroditism and pure gonadal dysgenesis. Lower serum AMH concentrations in otherwise healthy boys with cryptorchidism, who were compared with their age-matched counterparts with palpable testes, have been reported previously. AMH levels are higher in prepubertal patients with varicocele than in controls. This altered serum profile of AMH in boys with varicoceles may indicate an early abnormality in the regulation of the seminiferous epithelial function. Serum AMH is known to be valuable in assessing gonadal function. As compared to testing involving the administration of human chorionic gonadotropin, the measurement of AMH is more sensitive and equally specific. Measurement of AMH is very useful in young children, because serum gonadotropin concentrations in those who are agonadal are nondiagnostic in midchildhood and serum testosterone concentrations may fail to increase with provocative testing in children with abdominal testes.

## 1. Introduction

Anti-Müllerian hormone (AMH), also named Müllerian inhibiting substance (MIS), is a tissue-specific TGF-beta superfamily growth factor. AMH is secreted by immature Sertoli cells (SC) and is responsible for the regression of Müllerian ducts in the male fetus as part of the sexual differentiation process [1, 2]. AMH is also involved in testicular development and function [1, 2]. 

## 2. Physiology

### 2.1. Fetus

In the 7th week of gestation, the undifferentiated gonads differentiate into a testis in the XY embryo. Gonadal cells become segregated in two compartments: testicular cords and interstitial tissue. Testicular cords are composed by somatic SC and germ cells, surrounded by a basal membrane and peritubular cells. SC produce AMH and inhibin B. In early fetal life AMH expression is triggered by SOX9 gene, and enhanced by SF1 and WT1, independently of gonadotropic control [2, 3]. Later, FSH stimulates AMH production. In females, AMH is produced by the granulosa cells of primary and small antral follicles present in the ovaries from late fetal life throughout reproductive life [2].

Müllerian ducts regress in the male fetus during the 8th and 9th week of gestation through apoptosis and epithelial-mesenchymal transformation occurring in a cranial-to-caudal direction. By week 10, Müllerian ducts become insensitive to AMH [2, 4]. 

### 2.2. Neonate

The known transient increase of gonadotropins in the first hours after birth is followed by a sharp decrease as of the second day of life. By the 7th day of live, gonadotropins level is high again. Leydig's cell testosterone (T) production follows the LH surge, with a certain delay. Testosterone level is high during whole neonatal life. SC-specific peptides inhibin B and AMH are at their lowest levels in the first days after birth but increase after the first week, likely reflecting active SC proliferation [5]. This AMH increase is probably related to FSH-induced SC proliferation, and also to activation of AMH gene transcription through a pathway mediated by cAMP [6, 7]. AMH rises rapidly in concentration in boys during the first month, reaching a peak level at about 6 months of age, and then slowly declines during childhood, falling to low levels in puberty [8, 9]. 

In humans, androgens both induce spermatogenesis and repress AMH. Androgen receptor (AR) protein is present in Leydig and peritubular cells of fetal and neonatal human testis, but not in SC. The absence of AR expression in SC of fetal and neonatal human testis contributes to the lack of germ cell maturation and of AMH repression despite strong testicular testosterone biosynthesis.

AMH is undetectable (54%) or very low (95% CI: <2–16 pmol/L) in female infants.

### 2.3. Puberty

The pubertal decline in AMH results from gradual activation of the hypothalamic-pituitary-gonadal axis, and subsequent increase in intratesticular testosterone, rather than from the interaction between SC and spermatogenic cells [10, 11]. A functional AR appears to be essential for intratesticular testosterone-mediated AMH repression to occur in human SC [12]. The early increase in intratesticular testosterone level is responsible for the inhibition of AMH expression [13, 14]. The AR expression is first observed in the nuclei of few SC at the age of 5 months, and progressively increases to high levels of AR expression in more than 90% of SC nuclei by the age of 8 years. The presence of AR in boys older than 8 years old explains the early pubertal decline of AMH induced by intratesticular testosterone rise, despite the increase in FSH secretion [15].

The close relationship between AMH and inhibin B suggests that inhibin B is an indirect indicator of AR-mediated SC maturation [10]. 

While AMH expression is downregulated by meiotic germ cells, the expression of inhibin B *β*-subunit is dependent on the coexistence of spermatogenesis [2, 16, 17]. In men, suppression of spermatogenesis due to the lack of testosterone could stimulate AMH and inhibit inhibin B expression [16]. 

AMH expression and secretion by SC is regulated by inhibitory paracrine actions of intratesticular testosterone and neighbouring germ cells and by a stimulating hormonal effect of FSH. The effect of FSH on testicular AMH production might be due to a direct effect on AMH expression in each individual SC, a proliferative effect on SC, or both. The prepubertal testis is mainly composed of SC, which represent more than 75% of gonadal mass [15, 18] and are active [15, 19]. AMH determination may also be used to explore the functional reserve in response to FSH, because the spermatogenic potential of the testis is dependent on SC function [15, 20]. Basal and FSH-stimulated levels of AMH, might become a useful predictive marker of the spermatogenic response to gonadotropic treatment in young patients with hypogonadotropic hypogonadism (HH) [15, 21]. 

### 2.4. Adult

In the human adult testis, spermatogenesis is under control of FSH and LH. FSH acts directly on SC, LH induces testosterone production after Leydig cell stimulation. Intratesticular testosterone acts via a paracrine mechanism on AR expressed by target cells situated in the seminiferous tubules [2, 3, 5]. In contrast to the neonatal phase, SC in the adult human testis do express ARs. In adulthood, the action of androgens on the seminiferous tubules is essential for full, quantitatively normal spermatogenesis and fertility. Most evidence suggests that this effect is mediated through an effect on SC. The number of SC is directly associated with sperm-producing capacity, since each of these somatic cells can nurture only a limited number of developing spermatogenetic cells [2, 3, 5]. In adults, AMH type 2 receptors have been detected in SC, suggesting an autocrine effect. Also, paracrine effects of AMH on Leydig cells and adult germ cells were found, as AMH directly inhibits Leydig cell differentiation and steroidogenesis and might be involved in sperm motility [22]. In cases of infertility, in the absence of the androgen-inhibitory effect, FSH is able to enhance testicular AMH secretion in man [15].

AMH in the testis is secreted by SC both apically into seminiferous tubules and basally towards the interstitium and circulation. After puberty, AMH is released preferentially by the apical pole of the SC towards the lumen of the seminiferous tubules, resulting in higher concentrations in the seminal plasma than in the serum [23]. A reduced fertility potential is characterized by reduction in sperm motility, sperm concentration, testicular volume, and decreased inhibin B level [24]. Also, AMH concentration in seminal plasma in azoospermia is lower than in normal men [25]. The assay of seminal AMH may be considered as a tool for prediction of gonadotropin therapy outcome in hypogonadotropic hypogonadism, since its early increase may be a marker of good spermatogenic response [23].

## 3. Pathology

### 3.1. Persistent Müllerian Duct Syndrome

Ambiguous genitalia due to impaired androgen secretion or action may be a result of various conditions with low, normal, or high AMH level. Defects in AMH production and insensitivity to AMH due to receptor defects result in the persistent Müllerian duct syndrome (PMDS) [26]. AMH deficiency due to mutations in AMH gene represents an early-onset fetal hypogonadism with SC-specific dysfunction. Patients are otherwise normally virilised, indicating normal Leydig cell function [2]. The persistence of the uterus and Fallopian tubes is an unpredicted finding at surgery for hernia or cryptorchidism in boys. AMH is undetectable but inhibin B serum level and androgens are in the normal male range [26–28]. In the absence of cryptorchidism, testes contain germ cells but fertility is frequent [29]. PMDS should not be confused with testicular dysgenesis, where persistence of Müllerian derivatives is associated with external sexual ambiguity reflecting both AMH and androgen deficiency, that is, an early onset fetal hypogonadism with whole gonadal dysfunction [2].

### 3.2. Androgen Insensitivity Syndrome (AIS)

In the newborn, defects of androgen signaling in target organs result in an anatomical phenotype of Leydig cell-specific hypogonadism causing androgen deficiency. In the complete form of androgen insensitivity syndrome, female external genitalia with short vagina and the absence of uterus and Fallopian tubes reflect the lack of androgen action together with the normal AMH production [2, 26, 30]. However, amenorrhoea is permanent due to Müllerian regression in fetal life. In partial forms of AIS, ambiguous genitalia and Wolffian duct underdevelopment of various degrees are observed. The diagnosis is made by normal to high testosterone and AMH, and the absence of Müllerian derivatives [2, 26, 31].

AMH signals through two membrane receptors: the type 2 receptor (AMHR2), which binds to AMH, and a type 1 receptor involved in signal transduction [27]. Mutations in the genes encoding AMHR2 and the androgen receptor are associated with the specific hormone-resistance syndromes [31]. PMDS due to mutations in the AMHR2 provoke insensitivity to AMH owing to disrupted ligand binding, signal transduction, or cellular transport [32]. The anatomic picture does not differ from that observed in patients with AMH gene mutations. Serum AMH makes the distinction since it is normal or elevated in patients with AMH insensitivity [26, 27].

### 3.3. Congenital Hypogonadotropic Hypogonadism (HH)

 Congenital HH affects the development of SC [10]. Severe gonadotropin deficiency leads to a decreased number of SC and therefore low AMH and inhibin B levels [10, 33, 34]. Circulating AMH provides a potentially useful tool for differentiating congenital HH from constitutional delay of growth and puberty in males with delayed puberty [10, 33, 35]. The assessment of AMH, might predict the clinical onset of puberty without the need for repeated clinical examination or GnRH testing [10, 33]. The early pubertal increase in inhibin B is tightly coupled to a decrease in AMH and therefore may reflect androgen-mediated differentiation of SC. The low AMH levels in patients with congenital HH and prepubertal testis volume may be of clinical value in early diagnosis of this condition, and the very low AMH level in boys with profound congenital HH suggests impaired development of the SC population [10, 33]. In untreated hypogonadotropic hypogonadal men, FSH induces a gradual increase in AMH levels, when hCG is added—AMH secretion is suppressed [15]. In contrast, Boukari et al. observed that AMH levels were not reduced in the two adult patients with mild androgen insensitivity syndrome (MAIS), who received gonadotropin treatment [12]. This AMH response pattern in MAIS is similar to that recently reported in two HH neonates [36] receiving a similar treatment, indicating that in both cases the failure to repress AMH is related to the absence of functional AR expression in SC.

### 3.4. Cryptorchidism

A measurable value of AMH in a boy with bilateral cryptorchidism is predictive of undescended testes, while an undetectable value is highly suggestive of anorchia or ovaries, as would be the case in girls with female pseudohermaphroditism and pure gonadal dysgenesis [37]. Values above the normal range for women remain diagnostic of the presence of testicular tissue, except in women with granulosa-cell tumors or sex-cord tumors that secrete AMH [38, 39]. 

The first phase of typical testicular descent takes place between the 10th and 15th week of human gestation [40]. This occurrence is independent of androgen levels, as the process has been found to transpire in patients with complete androgen insensitivity, and may also be controlled by AMH and insulin-like hormone 3 (INSL3) [24, 41, 42]. INSL3 is secreted by the Leydig cells shortly after the onset of testicular development, and controls the thickening of the gubernaculum anchoring the testis to the inguinal region [43]. Disruption of the INSL3 gene in mice results in bilateral intra-abdominal testes [44, 45]. In humans on the other hand, it was found that only 1,9% of the cases of cryptorchidism were caused by INSL3 gene mutations, and that the mutations of the INSL3 receptor on the whole were uncommon [46, 47]. The second or inguinoscrotal phase of testicular descent occurs between 26th and 40th weeks of gestation [40]. During this phase, the testis migrates through the inguinal canal and across the pubic region to the scrotum. There is much clinical evidence, that shows reduced androgen action to be associated with undescended testes [24, 40–45]. 

Unilateral cryptorchidism carries an increased risk of infertility in adulthood. Up to 30% of men operated on in childhood for unilateral cryptorchidism are likely to be subfertile in later life [48–51]. Men who undergo an operation for bilateral cryptorchidism are more affected—up to 54% are infertile according to their semen and hormonal analysis [49, 52]. 

Lower serum AMH concentrations in otherwise healthy boys with cryptorchidism, who were compared with their age-matched counterparts with palpable testes, have been reported in several studies [53–55]. Some authors observed upward trend in AMH concentration one year after orchidopexy, but it was statistically insignificant [56]. In contrast, Aksglaede et al. did not find the difference in AMH concentrations between patients with Klinefelter Syndrome, with or without a history of cryptorchidism. The exception to this was noted in untreated patients, 10–14 years old, in whom the expected puberty decline in AMH tended to occur later than in the noncryptorchid patients of the same age [57]. 

### 3.5. Varicocele

In a large study of 124 boys with varicocele Trigo et al. showed that AMH levels were higher in prepubertal patients with varicocele than in controls. Similarly, inhibin B levels were higher in pubertal boys with varicoceles than in the controls [58]. This altered serum profile of gonadal hormones in boys with varicoceles may indicate an early abnormality in the regulation of the seminiferous epithelial function [58].

In another study, Goulis et al. measured peripheral vein and spermatic vein inhibin B and AMH concentrations. In peripheral vein inhibin B, concentrations in men with varicocele were lower as compared to controls, but there was no difference in AMH concentrations. Spermatic vein inhibin B concentrations in men with varicocele were higher compared to those of peripheral vein. On the contrary, spermatic vein AMH concentrations were lower compared to those from peripheral vein [59]. The clinical significance of AMH concentrations in peripheral and spermatic vein remains to be elucidated.

## 4. AMH as a Marker Evaluating Gonadal Function

Serum AMH is known to be valuable in assessing gonadal function [33, 60, 61]. As compared with testing involving the administration of human chorionic gonadotropin, the measurement of AMH is more sensitive and equally specific. Measurement of AMH is very useful in young children, because serum gonadotropin concentrations in those who are agonadal are nondiagnostic in midchildhood and serum testosterone concentrations may fail to increase with provocative testing in children with abdominal testes [33, 37, 62]. Testicular size and sperm density in adult men are positively correlated to germ-cell status in the testes in childhood [63, 64]. In prepubertal boys, low serum AMH correlates with small testes [47]. Early postnatal admission of recombinant human FSH is resulting in an increase of testicular size and elevation of serum AMH level [40]. 

## 5. Conclusion

AMH is one of the key factors conditioning the normal development of male genitals. Serum AMH determination is clinically valuable in assessing gonadal function. Basal and FSH-stimulated levels of AMH, might become a useful predictive marker of the spermatogenic response to gonadotropic treatment in young patients with hypogonadotropic hypogonadism. A measurable value of AMH in a boy with bilateral cryptorchidism is predictive of undescended testes, while an undetectable value is highly suggestive of anorchia or ovaries, as would be the case in girls with female pseudohermaphroditism and pure gonadal dysgenesis. AMH levels are higher in prepubertal patients with varicocele than in controls, which indicate an early abnormality in the regulation of the seminiferous epithelial function.
